# Boson–Fermion Algebraic Mapping in Second Quantization

**DOI:** 10.3390/e26121067

**Published:** 2024-12-08

**Authors:** Fabio Lingua, Diego Molina Peñafiel, Lucrezia Ravera, Sebastián Salgado

**Affiliations:** 1 Department of Applied Physics, KTH Royal Institute of Technology—KTH, SE-10691 Stockholm, Sweden; lingua@kth.se; 2 Instituto de Ciencias Exactas y Naturales, Facultad de Ciencias, Univesidad Arturo Prat—UNAP Avda., Arturo Prat 2120, Iquique 1110939, Chile; dimolina@unap.cl; 3 DISAT, Politecnico di Torino—PoliTo, Corso Duca degli Abruzzi 24, 10129 Torino, Italy; 4 Istituto Nazionale di Fisica Nucleare, Section of Torino—INFN, Via P. Giuria 1, 10125 Torino, Italy; 5 Grupo de Investigación en Física Teórica—GIFT, Universidad Católica De La Santísima Concepción, Concepción 4070129, Chile; 6 Instituto de Alta Investigación, Universidad de Tarapacá, Casilla 7D, Arica 1000000, Chile; ssalgador@gestion.uta.cl

**Keywords:** bosons and fermions, second quantization, Grassmann variables, gauge invariance

## Abstract

We present an algebraic method to derive the structure at the basis of the mapping of bosonic algebras of creation and annihilation operators into fermionic algebras, and vice versa, introducing a suitable identification between bosonic and fermionic generators. The algebraic structure thus obtained corresponds to a deformed Grassmann-type algebra, involving anticommuting Grassmann-type variables. The role played by the latter in implementing gauge invariance in second quantization within our procedure is then discussed. This discussion includes the application of the mapping to the case of the bosonic and fermionic harmonic oscillator Hamiltonians.

## 1. Introduction

Bosons and fermions have different characteristics and are distinguished by their intrinsic properties, particularly their spin properties and statistics. Bosons have integer spin, and obey Bose–Einstein statistics, meaning that multiple bosons can occupy the same quantum state simultaneously, which leads to phenomena such as Bose–Einstein condensation and the behavior of photons within a laser. Fermions obey Fermi–Dirac statistics and are subject to the Pauli exclusion principle, which states that no two fermions can occupy the same quantum state simultaneously.

In the quantum field theory (QFT), bosons are typically associated with fields that mediate interactions, and fermions are the building blocks of matter. In particle and nuclear physics, composite particles can be bosons if their constituent fermions pair up in such a way that their total spin is an integer (e.g., mesons are made of one quark and one antiquark), while composite particles can be fermions if their constituent fermions combine to give a half-integer spin (e.g., baryons, such as protons and neutrons, which are made of three quarks). Despite their differences, systems and theories of bosons and fermions can be mapped into each other. Under certain conditions and within well-defined regimes, one can express bosons in terms of fermions, and vice versa, allowing for a Boson–Fermion correspondence. For example, the bosonization technique [1,2,3,4,5,6] is a well-established analytic tool for investigating the low-energy regime of one-dimensional interacting fermionic systems, essentially involving linearizing the spectrum around the Fermi points, passing to the continuum limit, and finally expressing the fermionic operators in terms of bosonic fields. On the other hand, with the so-called fermionization technique, involving Jordan–Wigner transformations [7,8], it is possible to map spin and bosonic systems into fermionic ones; see [9] for a review of bosonization and fermionization. Furthermore, the Fock space for fermion fields can be identified with the Fock space for boson fields, provided that the overall numbers of internal degrees of freedom (d.o.f.) are the same. As a consequence, the respective free field Hamiltonian systems are equivalent, or, as is often said, dual. The underlying principles of connecting bosonic and fermionic descriptions through dualities are applicable across various domains in QFT, see, e.g., Ref. [10]. Another possible way to relate bosons and fermions may be through supersymmetry. One of the mathematical tools used to formulate and understand supersymmetry is Grassmann-type variables. In particular, supersymmetric theories can be formulated in a way that extends the concept of spacetime into a new structure called superspace, which includes both the usual spacetime coordinates and (anticommuting) Grassmann coordinates.

In this work, we present an algebraic approach to the mapping of the algebra of bosonic creation and annihilation operators (second quantization) into the algebra of fermionic operators, and vice versa, which allows us to systematically derive the algebraic structure underlying the mapping. Our procedure is based on the introduction of a proper identification criterion between bosonic and fermionic generators, derived from a Lie algebra expansion method known as S-expansion [11], and adapted for our purposes. The basis of the S-expansion consists of combining the inner multiplication law of a semigroup *S* with the structure constants of a Lie algebra G. The new Lie algebra is called an S-expanded algebra. From the physical point of view, several theories have been extensively studied using the S-expansion method, enabling numerous results in recent years, especially in the context of gravitational theories; see, e.g., [12,13,14,15,16,17,18,19,20,21,22,23,24,25,26,27,28,29]. The identification criterion we adopt here is reminiscent of the one described in [18]. However, the algebraic structure we obtain is not that of a semigroup. Rather, it corresponds to a graded (deformed) Grassmann algebra involving anticommuting variables. This is derived from the analysis of the (anti)commutation relations of the bosonic and fermionic algebras.

The remainder of this paper is organized as follows: In Section 2, we briefly review the identification criterion of the S-expansion. This criterion is then adapted to write the fermionic generators in terms of bosonic ones—converting, via the elements of the algebraic structure involved, the bosonic generators into fermionic ones—and vice versa. In Section 3, we describe our procedure and derive the algebraic structure underlying the mapping, both in the case where the identification preserves the creation and annihilation operations, and in the case where bosonic/fermionic creation operators are mapped into fermionic/bosonic annihilation operators. Moreover, we discuss the role played by the Grassmann-like variables underlying the mapping in realizing gauge invariance in second quantization within our procedure. In Section 4, we apply this to the case of the bosonic and fermionic harmonic oscillator Hamiltonians. Section 5 is devoted to the conclusion.

## 2. Review of the Identification Criterion

The basis of the so-called S-expansion involves combining the multiplication law of a *semigroup*, denoted by *S* (from which, the procedure takes its name), with the structure constants of a Lie algebra G [11]. The new (larger) Lie algebra obtained through this procedure is named S-expanded algebra and can be written as $G_{S}=S\times G$. Let us briefly review the original procedure.

Let $S=\{\lambda_{\alpha }\}$, α=1,…,N, be a finite (Abelian) semigroup. Let G be a Lie algebra with basis $\left\{T_{A}\right\}$ and structure constants ${C_{AB}}^{C}$, defined by the following commutation relations:

(1) $$\begin{array}{c} \left[T_{A},T_{B}\right]={C_{AB}}^{C}\;T_{C}. \end{array}$$

We introduce the expanded algebra $G_{S}=S\times G$, which we will call the “target" algebra, with basis $\left\{{\tilde{T}}_{A}\right\}$ and structure constants ${{\tilde{C}}_{AB}}^{C}$, defined by the following commutation relations:

(2) $$\begin{array}{c} \left[{\tilde{T}}_{A},{\tilde{T}}_{B}\right]={{\tilde{C}}_{AB}}^{C}{\tilde{T}}_{C}. \end{array}$$

In order for the semigroup *S* to be the one involved in the Lie algebra expansion $G_{S}=S\times G$ (or, the other way around, if one is interested in possibly finding a semigroup *S* relating G and $G_{S}$; see [18]), the following identification criterion between the S-expanded generators of the initial Lie algebra and the generators of the target one is introduced:

(3) $$\begin{array}{c} {\tilde{T}}_{A}=T_{\left(A,\alpha \right)}:=\lambda_{\alpha }{\tilde{T}}_{A}. \end{array}$$

In general, when performing this expansion procedure, one splits the semigroup *S* into subsets, which are considered to be “in resonance” (see [11,18] for details) with the G-partition in subspaces. In particular, within the S-expansion method, one has to perform the identification in Equation (3) for each element of the semigroup *S*, associating each element of each subset with the generators in the subspace related to the considered subset. Of course, the whole association–identification procedure does not affect the internal structure of the generators of the starting algebra.

Under the identification in Equation (3), the commutation relations between the generators of the target algebras are linked with the commutation relations of the S-expanded ones, as follows:

(4) $$\begin{array}{c} \left[{\tilde{T}}_{A},{\tilde{T}}_{B}\right]=\left[T_{\left(A,\alpha \right)},T_{\left(B,\beta \right)}\right]={K_{\alpha \beta }}^{\gamma }{C_{AB}}^{C}\;T_{\left(C,\gamma \right)}, \end{array}$$

or

(5) $$\begin{array}{c} \left[\lambda_{\alpha }T_{A},\lambda_{\beta }T_{B}\right]={K_{\alpha \beta }}^{\gamma }{C_{AB}}^{C}\;\lambda_{\gamma }T_{C}, \end{array}$$

where ${K_{\alpha \beta }}^{\gamma }$ is the so-called two-selector of the semigroup [11,18] (here, we use the symbol · to denote the product between the elements of the semigroup), defined as follows:

(6) $${K_{\alpha \beta }}^{\gamma }=\left\{\begin{array}{c} 1,\;\;\;\;\;\mathrm{when}\;\lambda_{\alpha }\cdot \lambda_{\beta }=\lambda_{\gamma },\\ 0,\;\;\;\;\;\mathrm{otherwise}. \end{array}\right.$$

For a given semigroup, *S*, the latter is known, meaning that when the multiplication laws of the semigroup are known, one can automatically write the associated two-selector. In the expansion procedure, its presence allows for avoiding deformations of the original algebra when implementing the S-expansion method itself.

Therefore, one can express the structure constants of the target algebra in terms of the two-selector and the structure constants of the original algebra, as follows:

(7) $$\begin{array}{c} {{\tilde{C}}_{AB}}^{C}={C_{\left(A,\alpha )(B,\beta \right)}}^{\left(C,\gamma \right)}:={K_{\alpha \beta }}^{\gamma }{C_{AB}}^{C}. \end{array}$$

On the other hand, considering Equation (5) and factorizing the elements of the semigroup *S* out of the commutators, the following relations can then be obtained as follows:

(8) $$\begin{array}{c} \left(\lambda_{\alpha }\cdot \lambda_{\beta }\right)\left[T_{A},T_{B}\right]={K_{\alpha \beta }}^{\gamma }{C_{AB}}^{C}\lambda_{\gamma }T_{C}. \end{array}$$

Comparing the commutation relation in Equation (8) with those of the starting algebra in Equation (1), one finds the following:

(9) $$\begin{array}{c} \lambda_{\alpha }\cdot \lambda_{\beta }={K_{\alpha \beta }}^{\gamma }\lambda_{\gamma }. \end{array}$$

If the semigroup is known, then this equation correctly reproduces its two-selector, that is, if we have the following:

(10) $$\begin{array}{c} \lambda_{\alpha }\cdot \lambda_{\beta }=\lambda_{\gamma }, \end{array}$$

then ${K_{\alpha \beta }}^{\gamma }=1$, and zero otherwise (cf. Equation (6)). On the other hand, this can be seen as a result of the multiplication table of the semigroup *S* involved in the mapping from a starting Lie algebra G to a target one $G_{S}$. This occurs when the structure constants of the original and target algebras are known entries. Indeed, this automatically informs on the two-selector appearing in Equation (7) and, consequently, as we can see from the above, on the multiplication laws of the semigroup *S* involved in the procedure. We remark that, in general, the latter is not uniquely determined, indicating that more semigroups may relate to the same two algebras. In this paper, we will simply inherit the identification criterion from the S-expansion procedure, without applying the rest of its machinery. For the sake of completeness and to aid the interested reader, Ref. [18] developed an analytic method to identify the semigroup, whenever it exists, involved in the S-expansion for transitioning to a target Lie (super)algebra from a starting one, after having properly chosen the partition over subspaces of the considered algebras (see also [19]). In this case, repeating the above procedure for all the commutation rules of the target algebra yields the multiplication rules between the elements of the semigroup *S*, i.e., its multiplication table.

In the following, we adopt and adapt the identification criterion in Equation (3) and derive the algebraic structure underlying the mapping between bosonic and fermionic algebras.

## 3. Algebraic Approach to the Boson–Fermion Mapping

We consider bosonic and fermionic creation and annihilation operators acting on modes, e.g., lattice sites. For the sake of clarity, we label bosonic modes with $I,J,\ldots =1,\ldots ,N_{B}$ and fermionic modes with $i,j,\ldots =1,\ldots ,N_{F}$.

We consider the bosonic algebra $G_{B}$ generated by $\{a_{I},a_{I}^{†}\}$, with $a_{I}$ and $a_{I}^{†}$ being the bosonic annihilation and creation operators, respectively, acting on mode *I*. They satisfy the following commutation relations:

(11) $$\begin{array}{cc} \; & \left[a_{I},a_{J}^{†}\right]=a_{I}a_{J}^{†}-a_{J}^{†}a_{I}=\delta_{IJ},\\ \; & \left[a_{I},a_{J}\right]=\left[a_{I}^{†},a_{J}^{†}\right]=0. \end{array}$$

As target algebra, we consider the fermionic algebra $G_{F}$ generated by $\{c_{i},c_{i}^{†}\}$, where $c_{i}$ and $c_{i}^{†}$ are fermionic annihilation and creation operators, respectively, acting on mode *i*. These generators satisfy the following anticommutation relations:

(12) $$\begin{array}{cc} \; & \left\{c_{i},c_{j}^{†}\right\}=\delta_{ij},\\ \; & \left\{c_{i},c_{j}\right\}=\left\{c_{i}^{†},c_{j}^{†}\right\}=0. \end{array}$$

Now, our goal is to find the algebraic structure linking the bosonic algebra $G_{B}$ and the fermionic algebra $G_{F}$. To this aim, we apply the identification criterion developed in the S-expansion context, adapted to our framework. As we shall see, two main different identifications are possible.

### 3.1. Mapping That Preserves the Creation/Annihilation Operation

We write the following identification relations between the fermionic and bosonic generators:

(13) $$\left\{\begin{array}{c} c_{i}={\lambda_{i}}^{I}a_{I},\\ c_{i}^{†}={\lambda_{i}}^{I†}a_{I}^{†}, \end{array}\right.$$

where ${\lambda_{i}}^{I}$ and ${\lambda_{i}}^{I†}$ are the elements of an algebraic structure Λ, to be determined in the following. (Note that these variables have nothing to do with the semigroup elements introduced in Section 2, other than the fact that they are involved in the implementation of the identification criterion at hand.) Notice that the λ variables carry mode labels, and the contracted indices in Equation (13) are summed over (summation is implied). There is no covariant/contravariant structure; the metric is Euclidean. Indices can be raised and lowered freely with a Kronecker delta. Uppercase (lowercase) refers to bosonic (fermionic) degrees of freedom. Indices on the left relate to the target algebra generators, and the ones on the right to generators of the starting algebra. We will use a · to denote the product between the λ and $\lambda^{†}$ variables.

We extract the algebraic properties of the algebraic structure Λ involved in the mapping—which, at this stage, involves consistently writing fermions in terms of bosons using the identification in Equation (13)—by analyzing the (anti)commutation relations of the fermionic and bosonic algebras. We start by considering the first anticommutation relation in Equation (12). Substituting the expressions in Equation (13) for the fermionic generators $c_{i}$ and $c_{j}^{†}$, we obtain the following:

(14) $$\begin{array}{cc} \left\{c_{i},c_{j}^{†}\right\} & =\delta_{ij}\\ \; & =\left\{{\lambda_{i}}^{I}a_{I},{\lambda_{j}}^{J†}a_{J}^{†}\right\}=\left({\lambda_{i}}^{I}\cdot {\lambda_{j}}^{J†}\right)a_{I}a_{J}^{†}+\left({\lambda_{j}}^{J†}\cdot {\lambda_{i}}^{I}\right)a_{J}^{†}a_{I}. \end{array}$$

Now, we see that by requiring a consistency relation to be fulfilled by λ, we obtain the following:

(15) $$\begin{array}{c} {\lambda_{i}}^{I}\cdot {\lambda_{j}}^{J†}=-{\lambda_{j}}^{J†}\cdot {\lambda_{i}}^{I}, \end{array}$$

and we obtain the following:

(16) $$\begin{array}{cc} \left\{c_{i},c_{j}^{†}\right\} & =\delta_{ij}\\ \; & ={\lambda_{i}}^{I}\cdot {\lambda_{j}}^{J†}\left(a_{I}a_{J}^{†}-a_{J}^{†}a_{I}\right)={\lambda_{i}}^{I}\cdot {\lambda_{j}}^{J†}\left[a_{I},a_{J}^{†}\right]={\lambda_{i}}^{I}\cdot {\lambda_{j}}^{J†}\delta_{IJ}, \end{array}$$

where in the last step we use the first commutation relation in Equation (11). Hence, we are left with the following relation:

(17) $$\begin{array}{c} {\lambda_{i}}^{I}\cdot \lambda_{jI}^{†}=\delta_{ij}. \end{array}$$

On the other hand, taking the anticommutation relation $\{c_{i},c_{j}\}=0$ and using the identification in Equation (13), we can write the following:

(18) $$\begin{array}{cc} \left\{c_{i},c_{j}\right\} & =0\\ \; & =\left\{{\lambda_{i}}^{I}a_{I},{\lambda_{j}}^{J}a_{J}\right\}={\lambda_{i}}^{I}\cdot {\lambda_{j}}^{J}\left[a_{I},a_{J}\right]=0, \end{array}$$

where we also introduce and exploit the consistency requirement, as follows:

(19) $$\begin{array}{c} {\lambda_{i}}^{I}\cdot {\lambda_{j}}^{J}=-{\lambda_{j}}^{J}\cdot {\lambda_{i}}^{I} \end{array}$$

and use the commutation relation $[a_{I},a_{J}]=0$. Analogously, considering the anticommutation relation $\{c_{i}^{†},c_{j}^{†}\}=0$, using the bosonic commutation relation $[a_{I}^{†},a_{J}^{†}]=0$, and implementing the identification in Equation (13) we obtain the following:

(20) $$\begin{array}{c} {\lambda_{i}}^{I†}\cdot {\lambda_{j}}^{J†}=-{\lambda_{j}}^{J†}\cdot {\lambda_{i}}^{I†}. \end{array}$$

Hence, we end up with the multiplication rules in Equations (15), (17), (19), and (20) between the Λ elements. The algebraic structure obtained is as follows:

(21) $$\begin{array}{cc} \; & \left\{{\lambda_{i}}^{I},{\lambda_{j}}^{J†}\right\}={\lambda_{i}}^{I}\cdot {\lambda_{j}}^{J†}+{\lambda_{j}}^{J†}\cdot {\lambda_{i}}^{I}=0,\\ \; & \left\{{\lambda_{i}}^{I},{\lambda_{j}}^{J}\right\}={\lambda_{i}}^{I}\cdot {\lambda_{j}}^{J}+{\lambda_{j}}^{J}\cdot {\lambda_{i}}^{I}=0,\\ \; & \left\{{\lambda_{i}}^{I†},{\lambda_{j}}^{J†}\right\}={\lambda_{i}}^{I†}\cdot {\lambda_{j}}^{J†}+{\lambda_{j}}^{J†}\cdot {\lambda_{i}}^{I†}=0, \end{array}$$

which corresponds to a graded Grassmann algebra, involving anticommuting Grassmann-type variables. The anticommuting elements ${\lambda_{i}}^{I}$ and ${\lambda_{i}}^{I†}$ are $Z_{2}$ odd-graded (fermionic) elements. The algebra they generate is $Z_{2}$-graded. Furthermore, one may refer to it as “deformed” Grassmann algebra, given that $\lambda ,\lambda^{†}$ obey the multiplication rule in Equation (17), which can be seen as an extra condition with respect to those appearing in standard Grassmann algebra. Note that the Pauli exclusion principle for fermions is still naturally satisfied—and it will also be so in the other mappings we will present, due to the fact that the original (anti)commutation relations are respected.

#### Inverse Mapping of Fermionic into Bosonic Operators

One can also consider the inverse mapping of fermions into bosons, by applying an analogous procedure, i.e., by writing an identification as follows:

(22) $$\left\{\begin{array}{c} a_{I}={\lambda_{I}}^{i}c_{i},\\ a_{I}^{†}={\lambda_{I}}^{i†}c_{i}^{†}, \end{array}\right.$$

with ${\lambda_{I}}^{i}$ and ${\lambda_{I}}^{i†}$ being the elements of the set involved in the mapping. In this case, from the analysis of the first commutation relation in Equation (11), we obtain the following:

(23) $$\begin{array}{cc} \left\{a_{I},a_{J}^{†}\right\} & =\delta_{IJ}\\ \; & =\left[{\lambda_{I}}^{i}c_{i},{\lambda_{J}}^{j†}c_{j}^{†}\right]=\left({\lambda_{I}}^{i}\cdot {\lambda_{J}}^{j†}\right)c_{i}c_{j}^{†}-\left({\lambda_{J}}^{j†}\cdot {\lambda_{I}}^{i}\right)c_{j}^{†}c_{i}\\ \; & ={\lambda_{I}}^{i}\cdot {\lambda_{J}}^{j†}\left(c_{i}c_{j}^{†}+c_{j}^{†}c_{i}\right)={\lambda_{I}}^{i}\cdot {\lambda_{J}}^{j†}\left\{c_{i},c_{j}^{†}\right\}={\lambda_{I}}^{i}\cdot {\lambda_{J}}^{j†}\delta_{ij}, \end{array}$$

where, in the second line, we implement the consistency requirement as follows:

(24) $$\begin{array}{c} {\lambda_{I}}^{i}\cdot {\lambda_{J}}^{j†}=-{\lambda_{J}}^{j†}\cdot {\lambda_{I}}^{i}, \end{array}$$

and make use of the anticommutation relation $\left\{c_{i},c_{j}\right\}=\delta_{ij}$. Hence, from Equation (23), we also obtain the following:

(25) $$\begin{array}{c} {\lambda_{I}}^{i}\cdot \lambda_{Ji}^{†}=\delta_{IJ}. \end{array}$$

On the other hand, we have the following:

(26) $$\begin{array}{cc} \left\{a_{I},a_{J}\right\} & =0\\ \; & =\left[{\lambda_{I}}^{i}c_{i},{\lambda_{J}}^{j}c_{j}\right]=\left({\lambda_{I}}^{i}\cdot {\lambda_{J}}^{j}\right)c_{i}c_{j}-\left({\lambda_{J}}^{j}\cdot {\lambda_{I}}^{i}\right)c_{j}c_{i}\\ \; & ={\lambda_{I}}^{i}\cdot {\lambda_{J}}^{j}\left(c_{i}c_{j}+c_{j}c_{i}\right)={\lambda_{I}}^{i}\cdot {\lambda_{J}}^{j}\left\{c_{i},c_{j}\right\}=0, \end{array}$$

where, for consistency, we have the following:

(27) $$\begin{array}{c} {\lambda_{I}}^{i}\cdot {\lambda_{J}}^{j}=-{\lambda_{J}}^{j}\cdot {\lambda_{I}}^{i}. \end{array}$$

Analogously, from the analysis of $[a_{I}^{†},a_{J}^{†}]=0$, via $\{c_{i}^{†},c_{j}^{†}\}=0$, for consistency, we can derive the multiplication rule as follows:

(28) $$\begin{array}{c} {\lambda_{I}}^{i†}\cdot {\lambda_{J}}^{j†}=-{\lambda_{J}}^{j†}\cdot {\lambda_{I}}^{i†}. \end{array}$$

Therefore, in the case of inverse mapping, we end up with the multiplication rules in Equations (24), (25), (27), and (28) between the elements of the set underlying the mapping. Also in this case, the algebraic structure obtained corresponds to graded, deformed Grassmann-type algebra, involving anticommuting Grassmann-type variables, and reads as follows:

(29) $$\begin{array}{cc} \; & \{{\lambda_{I}}^{i},{\lambda_{J}}^{j†}\}=0,\\ \; & \{{\lambda_{I}}^{i},{\lambda_{J}}^{j}\}=0,\\ \; & \{{\lambda_{I}}^{i†},{\lambda_{J}}^{j†}\}=0, \end{array}$$

together with the extra relation in Equation (25).

Considering the identifications in Equations (13) and (22) together, we may write the following:

(30) $$\begin{array}{cc} a_{J}={\lambda_{J}}^{i}c_{i}={\lambda_{J}}^{i}{\lambda_{i}}^{I}a_{I}=\left({\lambda_{J}}^{i}\cdot {\lambda_{i}}^{I}\right)a_{I}\;\Rightarrow \; & {\lambda_{J}}^{i}\cdot {\lambda_{i}}^{I}={\delta_{J}}^{I}, \end{array}$$

(31) $$\begin{array}{cc} a_{J}^{†}={\lambda_{J}}^{i†}c_{i}^{†}={\lambda_{J}}^{i†}{\lambda_{i}}^{I†}a_{I}^{†}=\left({\lambda_{J}}^{i†}\cdot {\lambda_{i}}^{I†}\right)a_{I}^{†}\;\Rightarrow \; & {\lambda_{J}}^{i†}\cdot {\lambda_{i}}^{I†}={\delta_{J}}^{I}, \end{array}$$

(32) $$\begin{array}{cc} c_{j}={\lambda_{j}}^{I}a_{I}={\lambda_{j}}^{I}{\lambda_{I}}^{i}c_{i}=\left({\lambda_{j}}^{I}\cdot {\lambda_{I}}^{i}\right)c_{i}\;\Rightarrow \; & {\lambda_{j}}^{I}\cdot {\lambda_{I}}^{i}={\delta_{j}}^{i}, \end{array}$$

(33) $$\begin{array}{cc} c_{j}^{†}={\lambda_{j}}^{I†}a_{I}^{†}={\lambda_{j}}^{I†}{\lambda_{I}}^{i†}c_{i}^{†}=\left({\lambda_{j}}^{I†}\cdot {\lambda_{I}}^{i†}\right)c_{i}^{†}\;\Rightarrow \; & {\lambda_{j}}^{I†}\cdot {\lambda_{I}}^{i†}={\delta_{j}}^{i}, \end{array}$$

ending up with (multiplicative inverse) relations between the ${\lambda_{I}}^{i},{\lambda_{I}}^{i†}$ and the ${\lambda_{i}}^{I},{\lambda_{i}}^{I†}$ elements.

### 3.2. Mapping That Exchanges the Creation and Annihilation Operations

In contrast to Equation (13), another identification that one may consider is as follows:

(34) $$\left\{\begin{array}{c} c_{i}={\lambda_{i}}^{I†}a_{I}^{†},\\ c_{i}^{†}={\lambda_{i}}^{I}a_{I}, \end{array}\right.$$

where the creation/annihilation of bosons is translated into the corresponding annihilation/creation of fermions.

From the study of the first anticommutation relation in Equation (12), we obtain the following:

(35) $$\begin{array}{cc} \left\{c_{i},c_{j}^{†}\right\} & =\delta_{ij}\\ \; & =\left\{{\lambda_{i}}^{I†}a_{I}^{†},{\lambda_{j}}^{J}a_{J}\right\}=\left({\lambda_{i}}^{I†}\cdot {\lambda_{j}}^{J}\right)a_{I}^{†}a_{J}+\left({\lambda_{j}}^{J}\cdot {\lambda_{i}}^{I†}\right)a_{J}a_{I}^{†}\\ \; & ={\lambda_{i}}^{I†}\cdot {\lambda_{j}}^{J}\left(a_{I}^{†}a_{J}-a_{J}a_{I}^{†}\right)=-{\lambda_{i}}^{I†}\cdot {\lambda_{j}}^{J}\left[a_{J},a_{I}^{†}\right]={\lambda_{j}}^{J}\cdot {\lambda_{i}}^{I†}\delta_{JI}, \end{array}$$

where, for consistency, we obtain the following relation:

(36) $$\begin{array}{c} {\lambda_{i}}^{I†}\cdot {\lambda_{j}}^{J}=-{\lambda_{j}}^{J}\cdot {\lambda_{i}}^{I†} \end{array}$$

to hold (which is reminiscent of the relation in Equation (15) previously obtained for the λ and $\lambda^{†}$ elements), where we also use the first commutation relation in Equation (11). Therefore, we end up with the following relation:

(37) $$\begin{array}{c} {\lambda_{j}}^{I}\cdot {\lambda_{iI}}^{†}=\delta_{ij} \end{array}$$

between the λ and $\lambda^{†}$ (analogous to the relation in Equation (17) previously obtained for the λ and $\lambda^{†}$). Then, considering the other anticommutation relations in the fermionic algebra we obtain the following:

(38) $$\begin{array}{cc} \left\{c_{i},c_{j}\right\} & =0\\ \; & =\left\{{\lambda_{i}}^{I†}a_{I}^{†},{\lambda_{j}}^{J†}a_{J}^{†}\right\}={\lambda_{i}}^{I†}\cdot {\lambda_{j}}^{J†}\left[a_{I}^{†},a_{J}^{†}\right]=0 \end{array}$$

and

(39) $$\begin{array}{cc} \left\{c_{i}^{†},c_{j}^{†}\right\} & =0\\ \; & =\left\{{\lambda_{i}}^{I}a_{I},{\lambda_{j}}^{J}a_{J}\right\}={\lambda_{i}}^{I}\cdot {\lambda_{j}}^{J}\left[a_{I},a_{J}\right]=0, \end{array}$$

where we also introduce and exploit the consistency requirements, as follows:

(40) $$\begin{array}{c} {\lambda_{i}}^{I†}\cdot {\lambda_{j}}^{J†}=-{\lambda_{j}}^{J†}\cdot {\lambda_{i}}^{I†} \end{array}$$

and

(41) $$\begin{array}{c} {\lambda_{i}}^{I}\cdot {\lambda_{j}}^{J}=-{\lambda_{j}}^{J}\cdot {\lambda_{i}}^{I}, \end{array}$$

respectively, analogous to Equations (20) and (19) previously obtained for the $\lambda^{†}$ and λ variables. Hence, the algebraic structure underlying the mapping that converts the creation operation into the annihilation one, and vice versa, is the same as we have obtained in the case of the mapping that preserves the creation/annihilation operation, namely, a deformed Grassmann-type algebra (due to Equation (37)) involving anticommuting Grassmann-type variables.

#### Inverse Mapping of Fermionic into Bosonic Operators

In a similar way, we may now introduce the following identification:

(42) $$\left\{\begin{array}{c} a_{I}={\lambda_{I}}^{i†}c_{i}^{†},\\ a_{I}^{†}={\lambda_{I}}^{i}c_{i}, \end{array}\right.$$

translating the creation/annihilation of fermions into the annihilation/creation of bosons, which is the inverse mapping of Equation (34). Thus, from the analysis of the bosonic algebra, we obtain the following:

(43) $$\begin{array}{cc} \left\{a_{I},a_{J}^{†}\right\} & =\delta_{IJ}\\ \; & =\left[{\lambda_{I}}^{i†}c_{i}^{†},{\lambda_{J}}^{j}c_{j}\right]=\left({\lambda_{I}}^{i†}\cdot {\lambda_{J}}^{j}\right)c_{i}^{†}c_{j}-\left({\lambda_{J}}^{j}\cdot {\lambda_{I}}^{i†}\right)c_{j}c_{i}^{†}\\ \; & ={\lambda_{I}}^{i†}\cdot {\lambda_{J}}^{j}\left(c_{j}c_{i}^{†}+c_{i}^{†}c_{j}\right)={\lambda_{I}}^{i†}\cdot {\lambda_{J}}^{j}\left\{c_{j},c_{i}^{†}\right\}={\lambda_{I}}^{i†}\cdot {\lambda_{J}}^{j}\delta_{ji}, \end{array}$$

where we also implement the consistency requirement, as follows:

(44) $$\begin{array}{c} {\lambda_{I}}^{i}\cdot {\lambda_{J}}^{j†}=-{\lambda_{J}}^{j†}\cdot {\lambda_{I}}^{i}, \end{array}$$

analogous to the relation in Equation (24) previously written for λ and $\lambda^{†}$. Therefore, from Equation (43), we are left with the following relation:

(45) $$\begin{array}{c} {\lambda_{I}}^{i†}\cdot \lambda_{Ji}=\delta_{IJ}\;\Rightarrow \;\lambda_{Ji}\cdot {\lambda_{I}}^{i†}=-\delta_{IJ}. \end{array}$$

Moreover, we have the following:

(46) $$\begin{array}{cc} \left\{a_{I},a_{J}\right\} & =0\\ \; & =\left[{\lambda_{I}}^{i†}c_{i}^{†},{\lambda_{J}}^{j†}c_{j}^{†}\right]=\left({\lambda_{I}}^{i†}\cdot {\lambda_{J}}^{j†}\right)c_{i}^{†}c_{j}^{†}-\left({\lambda_{J}}^{j†}\cdot {\lambda_{I}}^{i†}\right)c_{j}^{†}c_{i}^{†}\\ \; & ={\lambda_{I}}^{i†}\cdot {\lambda_{J}}^{j†}\left(c_{i}^{†}c_{j}^{†}+c_{j}^{†}c_{i}^{†}\right)={\lambda_{I}}^{i†}\cdot {\lambda_{J}}^{j†}\left\{c_{i}^{†},c_{j}^{†}\right\}=0, \end{array}$$

where we use the following:

(47) $$\begin{array}{c} {\lambda_{I}}^{i†}\cdot {\lambda_{J}}^{j†}=-{\lambda_{J}}^{j†}\cdot {\lambda_{I}}^{i†}, \end{array}$$

reminiscent of Equation (28) among the $\lambda^{†}$ variables. Analogously, from the analysis of $[a_{I}^{†},a_{J}^{†}]=0$, via $\{c_{i},c_{j}\}=0$, we derive the multiplication rule, as follows:

(48) $$\begin{array}{c} {\lambda_{I}}^{i}\cdot {\lambda_{J}}^{j}=-{\lambda_{J}}^{j}\cdot {\lambda_{I}}^{i}, \end{array}$$

reminiscent of Equation (27) for λ. Hence, the algebraic structure obtained for the λ and $\lambda^{†}$ is, again, a graded (deformed) Grassmann algebra, with the deformation due to the extra relation in Equation (45).

Notice that, as previously observed in the case of the mapping that preserves the creation/annihilation operation, one may now consider Equations (34) and (42) together and write the following:

(49) $$\begin{array}{cc} a_{J}={\lambda_{J}}^{i†}c_{i}^{†}={\lambda_{J}}^{i†}{\lambda_{i}}^{I}a_{I}=\left({\lambda_{J}}^{i†}\cdot {\lambda_{i}}^{I}\right)a_{I}\;\Rightarrow \; & {\lambda_{J}}^{i†}\cdot {\lambda_{i}}^{I}={\delta_{J}}^{I}, \end{array}$$

(50) $$\begin{array}{cc} a_{J}^{†}={\lambda_{J}}^{i}c_{i}={\lambda_{J}}^{i}{\lambda_{i}}^{I†}a_{I}^{†}=\left({\lambda_{J}}^{i}\cdot {\lambda_{i}}^{I†}\right)a_{I}^{†}\;\Rightarrow \; & {\lambda_{J}}^{i}\cdot {\lambda_{i}}^{I†}={\delta_{J}}^{I}, \end{array}$$

(51) $$\begin{array}{cc} c_{j}={\lambda_{j}}^{I†}a_{I}^{†}={\lambda_{j}}^{I†}{\lambda_{I}}^{i}c_{i}=\left({\lambda_{j}}^{I†}\cdot {\lambda_{I}}^{i}\right)c_{i}\;\Rightarrow \; & {\lambda_{j}}^{I†}\cdot {\lambda_{I}}^{i}={\delta_{j}}^{i}, \end{array}$$

(52) $$\begin{array}{cc} c_{j}^{†}={\lambda_{j}}^{I}a_{I}={\lambda_{j}}^{I}{\lambda_{I}}^{i†}c_{i}^{†}=\left({\lambda_{j}}^{I}\cdot {\lambda_{I}}^{i†}\right)c_{i}^{†}\;\Rightarrow \; & {\lambda_{j}}^{I}\cdot {\lambda_{I}}^{i†}={\delta_{j}}^{i}, \end{array}$$

obtaining (multiplicative inverse) relations between the ${\lambda_{I}}^{i},{\lambda_{I}}^{i†}$ and the ${\lambda_{i}}^{I},{\lambda_{i}}^{I†}$ elements.

### 3.3. Gauge Invariance in Second Quantization and Role of the Grassmann-Type Variables

Let us conclude this section with an observation on gauge invariance and symmetry reduction in our context. In second quantization, gauge transformations correspond to phase transformations of the creation and annihilation operators for fermions/bosons. These transformations reflect the invariance of the system under local or global phase changes; in particular, the latter leave physical observables, such as occupation numbers, unchanged. For fermionic creation and annihilation operators, for a global U(1) gauge transformation, the operators transform as follows:

(53) $$\begin{array}{c} c_{i}\to c_{i}^{\prime }=e^{i\theta }c_{i},\;c_{i}^{†}\to {c^{\prime }}_{i}^{†}=e^{-i\theta }c_{i}^{†}, \end{array}$$

where θ is the phase angle of the transformation. This kind of transformation corresponds to a global symmetry where the phase factor is the same for all operators. Analogously, for bosonic creation and annihilation operators, one may write the following:

(54) $$\begin{array}{c} a_{I}\to a_{I}^{\prime }=e^{i\theta }a_{I},\;a_{I}^{†}\to {a^{\prime }}_{I}^{†}=e^{-i\theta }a_{I}^{†}. \end{array}$$

On the other hand, for a *local* U(1) gauge transformation, the phase factor can depend on the “position" (on the mode index, e.g., the site of a lattice model). While global gauge transformations leave physical observables unchanged because they apply a constant phase to all states in the system, local gauge transformations can affect observables unless they are compensated by other modifications in the system (such as the introduction of gauge fields). (Under a local phase rotation, a gauge field does not transform multiplicatively—in the Abelian case, it transforms affinely. Therefore, to establish our Boson-Fermion correspondence at the level of creation/annihilation operator algebras after introducing the gauge field modes as independent degrees of freedom, one should introduce an algebraic structure that transforms purely bosonic modes into fermionic modes in a “non-multiplicative" manner. This could be the case in a super-algebraic or supersymmetric framework, where these transformations are part of a more complex algebraic operation than simple multiplication. We note that this does not necessarily involve (standard) supersymmetry. Matching the number of bosonic and fermionic degrees of freedom is not necessarily required [30]. Although not often emphasized in the literature, it is crucial to recognize that supersymmetry is not a gauge symmetry. This distinction is more evident and better appreciated in the geometric approach (i.e., *rheonomic*) to supersymmetric theories in superspace [31].) Let us denote the aforementioned position or mode dependence by $\theta_{i}$ (or $\theta_{I}$), reflecting local symmetry. The fermionic creation and annihilation operators then transform as follows:

(55) $$\begin{array}{c} c_{i}\to c_{i}^{\prime }=e^{i\theta_{i}}c_{i},\;c_{i}^{†}\to {c^{\prime }}_{i}^{†}=e^{-i\theta_{i}}c_{i}^{†}, \end{array}$$

while for bosons we have the following:

(56) $$\begin{array}{c} a_{I}\to a_{I}^{\prime }=e^{i\theta_{I}}a_{I},\;a_{I}^{†}\to {a^{\prime }}_{I}^{†}=e^{-i\theta_{I}}a_{I}^{†}. \end{array}$$

Taking this into account and exploiting the identifications in Equations (13) and (22) that induce the previously discussed mappings, we analyze the $\theta_{I}$ gauge transformations of the different λ and $\lambda^{†}$ elements to be as follows:

(57) $$\begin{array}{c} {\lambda_{i}}^{I}\to {\lambda_{i}^{\prime }}^{I}=e^{-i\theta_{I}}{\lambda_{i}}^{I},\;{\lambda_{i}}^{I†}\to {\lambda_{i}^{\prime }}^{I†}=e^{i\theta_{I}}{\lambda_{i}}^{I†},\;{\lambda_{I}}^{i}\to {\lambda_{I}^{\prime }}^{i}=e^{i\theta_{I}}{\lambda_{I}}^{i},\;{\lambda_{I}}^{i†}\to {\lambda_{I}^{\prime }}^{i†}=e^{-i\theta_{I}}{\lambda_{I}}^{i†}, \end{array}$$

while the $\theta_{i}$ gauge transformations are as follows:

(58) $$\begin{array}{c} {\lambda_{i}}^{I}\to {\lambda_{i}^{\prime }}^{I}=e^{i\theta_{i}}{\lambda_{i}}^{I},\;{\lambda_{i}}^{I†}\to {\lambda_{i}^{\prime }}^{I†}=e^{-i\theta_{i}}{\lambda_{i}}^{I†},\;{\lambda_{I}}^{i}\to {\lambda_{I}^{\prime }}^{i}=e^{-i\theta_{i}}{\lambda_{I}}^{i},\;{\lambda_{I}}^{i†}\to {\lambda_{I}^{\prime }}^{i†}=e^{i\theta_{i}}{\lambda_{I}}^{i†}. \end{array}$$

Correspondingly, the creation and annihilation operators $c_{i}^{†},c_{i}$ and $a_{I}^{†},a_{I}$ can be seen as composite objects, invariant under (part of) the gauge symmetry. In other words, omitting mode labels to lighten the notation, one may formally write the following:

(59) $$\begin{array}{c} a^{\lambda }:=\lambda^{-1}a=\lambda^{-1}\left(\lambda c\right)=c,\;a^{†\lambda^{†}}:=\lambda^{†-1}a^{†}=\lambda^{†-1}\left(\lambda^{†}c^{†}\right)=c^{†}, \end{array}$$

and, considering the λ and $\lambda^{†}$ variables involved in the inverse mapping, we have the following:

(60) $$\begin{array}{c} c^{\lambda }:=\lambda^{-1}a=\lambda^{-1}\left(\lambda a\right)=a,\;c^{†\lambda^{†}}:=\lambda^{†-1}a^{†}=\lambda^{†-1}\left(\lambda^{†}a^{†}\right)=a^{†}. \end{array}$$

This allows us to interpret the creation and annihilation operators for bosons (fermions) as dressed fermionic (bosonic) operators. This can be considered *self-dressing*, as it is extracted from the operators themselves. The dressing is implemented by Grassmann-type variables $\lambda ,\lambda^{†}$, which one may refer to as *dressing Grassmannian variables*. As shown in Equations (57) and (58), under local gauge transformations, the latter phases are adjusted to cancel the phase change in the bosonic/fermionic operators, thus creating operators that are gauge-invariant under the symmetry that has been reduced. Note that, depending on the type of system being considered, i.e., whether it is fermionic or bosonic, part of the above-mentioned gauge symmetry transformations of $\lambda ,\lambda^{†}$ can be interpreted as residual gauge symmetries of the (partially) dressed operators.

The same arguments above can be applied, in an analogous way, to the mappings that exchange the creation and annihilation operations. In this case, taking into account Equations (55) and (56), and considering the mappings Equations (34)/(42), we obtain the following:

(61) $$\begin{array}{c} {\lambda_{i}}^{I}\to {\lambda_{i}^{\prime }}^{I}=e^{-i\theta_{I}}{\lambda_{i}}^{I},\;{\lambda_{i}}^{I†}\to {\lambda_{i}^{\prime }}^{I†}=e^{i\theta_{I}}{\lambda_{i}}^{I†},\;{\lambda_{I}}^{i}\to {\lambda_{I}^{\prime }}^{i}=e^{-i\theta_{I}}{\lambda_{I}}^{i},\;{\lambda_{I}}^{i†}\to {\lambda_{I}^{\prime }}^{i†}=e^{i\theta_{I}}{\lambda_{I}}^{i†}, \end{array}$$

and

(62) $$\begin{array}{c} {\lambda_{i}}^{I}\to {\lambda_{i}^{\prime }}^{I}=e^{-i\theta_{i}}{\lambda_{i}}^{I},\;{\lambda_{i}}^{I†}\to {\lambda_{i}^{\prime }}^{I†}=e^{i\theta_{i}}{\lambda_{i}}^{I†},\;{\lambda_{I}}^{i}\to {\lambda_{I}^{\prime }}^{i}=e^{-i\theta_{i}}{\lambda_{I}}^{i},\;{\lambda_{I}}^{i†}\to {\lambda_{I}^{\prime }}^{i†}=e^{i\theta_{i}}{\lambda_{I}}^{i†}, \end{array}$$

under $\theta_{I}$ and $\theta_{i}$ gauge transformations, respectively. Therefore, on the one hand, one may formally write the following:

(63) $$\begin{array}{c} a^{\lambda^{†}}:=\lambda^{†-1}a=\lambda^{†-1}\left(\lambda^{†}c^{†}\right)=c^{†},\;a^{†\lambda }:=\lambda^{-1}a^{†}=\lambda^{-1}\left(\lambda c\right)=c, \end{array}$$

and, on the other hand, we have the following:

(64) $$\begin{array}{c} c^{\lambda^{†}}:=\lambda^{†-1}a^{†}=\lambda^{†-1}\left(\lambda^{†}a^{†}\right)=a^{†},\;c^{†\lambda }:=\lambda^{-1}a=\lambda^{-1}\left(\lambda a\right)=a, \end{array}$$

considering the λ and $\lambda^{†}$ of the inverse mapping.

## 4. Application to the Hamiltonians of the Bosonic/Fermionic Harmonic Oscillators

We will now apply the algebraic mappings and reasoning presented above to the case of the (free) Hamiltonian of the bosonic and fermionic harmonic oscillators. Here, we explicitly apply the mapping that preserves the creation/annihilation operation; the same reasoning can be performed for the mapping that exchanges the creation/annihilation operation, leading, in a straightforward way, to algebraically analogous results. The Hamiltonian of the bosonic harmonic oscillator is as follows:

(65) $$\begin{array}{c} H_{B}=\frac{\hbar \omega }{2}\left\{a_{I}^{†},a_{I}\right\}=\frac{\hbar \omega }{2}\left(a_{I}^{†}a_{I}+a_{I}a_{I}^{†}\right)=\hbar \omega \left(a_{I}^{†}a_{I}+\frac{1}{2}\right), \end{array}$$

where summation over *I* is implied. Applying the identification in Equation (22) to $H_{B}$ and working down with the induced algebraic mapping, exploiting the previously derived relations between the associated variables $\lambda ,\lambda^{†}$, we obtain the following:

(66) $$\begin{array}{c} H_{B}=\frac{\hbar \omega }{2}{\lambda_{I}}^{i†}\cdot {\lambda_{I}}^{j}\left(c_{i}^{†}c_{j}-c_{j}c_{i}^{†}\right)=\frac{\hbar \omega }{2}{\lambda_{I}}^{i†}\cdot {\lambda_{I}}^{j}\left[c_{i}^{†},c_{j}\right]. \end{array}$$

In other words, through our procedure, one can consistently derive the Hamiltonian $H_{B}$ starting from the commutator $\left[c_{i}^{†},c_{j}\right]$ between fermionic operators. By using $\lambda ,\lambda^{†}$, we construct an object that remains invariant under $\theta_{i}$ gauge transformations—thus, this symmetry is reduced—and is also invariant under $\theta_{I}$ gauge transformations by design (as expected for $H_{B}$).

In a similar way, one can consider the Hamiltonian of the so-called fermionic harmonic oscillator, as follows:

(67) $$\begin{array}{c} H_{F}=\frac{\hbar \omega }{2}\left[c_{i}^{†},c_{i}\right], \end{array}$$

and by applying the identification in Equation (13) and using the relations between the $\lambda ,\lambda^{†}$ variables underlying the associated mapping, we can write the following:

(68) $$\begin{array}{c} H_{F}=\frac{\hbar \omega }{2}{\lambda_{i}}^{I†}\cdot {\lambda_{i}}^{J}\left(a_{I}^{†}a_{J}+a_{J}a_{I}^{†}\right)=\frac{\hbar \omega }{2}{\lambda_{i}}^{I†}\cdot {\lambda_{i}}^{J}\left\{a_{I}^{†},a_{J}\right\}. \end{array}$$

Therefore, we can see that the Hamiltonian $H_{F}$ can be derived from the anticommutator $\left\{a_{I}^{†},a_{J}\right\}$ between bosonic operators. This derivation is facilitated by constructing, via the Grassmann-type variables $\lambda ,\lambda^{†}$ appearing in Equation (13), an object that is invariant under $\theta_{I}$ gauge transformations. By design, it is also invariant under $\theta_{i}$ transformations, as expected for $H_{F}$.

Under the above perspective, the bosonic (fermionic) Hamiltonian $H_{B}$ ($H_{F}$) objects can be seen as dressed objects, derived from bare commutation (anticommutation) relations between creation and annihilation operators.

## 5. Conclusions

In this work, we present an algebraic approach to the mapping of Lie algebras $G_{B}$ of bosonic creation and annihilation operators into algebras $G_{F}$ of fermionic creation and annihilation operators, and vice versa.

We introduce a specific identification criterion, inherited from a Lie algebra expansion method, known as the S-expansion, and adapted to our purposes, between the bosonic and fermionic generators. We then use the (anti)commutation relations of the bosonic and fermionic algebras to determine the algebraic structure underlying the mapping. The latter corresponds to a graded, deformed Grassmann-type algebra, involving anticommuting Grassmann-type variables. The deformation is due to an extra relation among the elements of the algebra necessary for the consistency of the design based on the identification criterion adopted case by case. We present different mappings, all based on a Grassmann-type algebra, which either preserve the creation/annihilation operations or exchange them. In both cases, we analyzed the mapping of bosonic operators to fermionic operators and their inverses.

We then discuss the role played by the Grassmann-type variables concerning gauge invariance in second quantization. We found that, within our procedure for the various mappings, the bosonic/fermionic creation and annihilation operators can be seen as dressed operators, where the dressing is provided by Grassmann-type variables underlying the mappings, which can be interpreted as *dressing Grassmannian variables*. Under local gauge transformations, the latter variables transform in such a way as to compensate for changes in the bosonic/fermionic operators. The resulting composite operators are gauge-invariant under the symmetry that has been “reduced" (i.e., that is no longer manifest). Notice that, under this perspective, both bosonic and fermionic operators can be interpreted as “composite", given by the proper dressing via Grassmann-type variables of fermionic and bosonic operators, respectively. These kinds of “dressing variables" might also be extracted from the physical content of the model by considering, e.g., the polar decompositions of (complex) eigenvalues, which separate the magnitude and phase. We leave such analysis to future investigations.

We also provide an example of the application to the case of the Hamiltonians of the bosonic and fermionic harmonic oscillators, showing that they can be seen as dressed objects. These are derived from the bare commutation (for fermionic operators) and anticommutation (for bosonic operators) relations between the creation and annihilation operators. Future perspectives include the application/extension of the algebraic method developed here within a field-theoretic context and the analysis of various Hamiltonian systems (e.g., Hubbard models) by using the proposed mappings, considering on-site and in-site interaction terms. One may also consider extending our setup to the case of non-Abelian gauge or global rotations at the field-theoretic level. This extension would preserve the core approach but introduce added complexity due to the structure of non-Abelian symmetries. Specifically, the dressing process would need to accommodate the richer algebraic relationships inherent in these symmetries. Such an extension could provide new insights into the interplay between fermionic and bosonic degrees of freedom under non-Abelian symmetry constraints.

Finally, it would be interesting to see if and how our approach actually makes contact with supergeometry and/or supersymmetry, particularly concerning how we recover gauge invariance, especially in the context of the so-called *unconventional supersymmetry* (Ususy) [32,33,34,35], which has been shown to play a relevant role in the design of analog (supergravity) models, providing a macroscopic description of the electronic properties of graphene-like materials. Here, supersymmetry is not manifest, but the descriptions of these kinds of systems still derive from a supergeometry setup and exploit the so-called *matter Ansatz*, constructed with a bosonic field and a spin-1/2 fermion field, invariant under Nieh-Yan-Weyl transformations [36]. Models exhibiting Ususy do not require the matching of bosonic and fermionic degrees of freedom typical of supersymmetric theories; they involve dynamical spin-1/2 fermion fields and are particularly appealing because they are based on a Weyl-invariant action. Future investigations in this direction will be carried out from a field-theoretical perspective, at both the classical and quantum levels.

## Data Availability

No new data were created or analyzed in this study.

## References

[1] Coleman S.R. (1975). The Quantum Sine-Gordon Equation as the Massive Thirring Model. Phys. Rev. D.

[2] Emery V.J., Devreese J.T., Evrard R.P., van Doren V.E. (1979). Theory of the one-dimensional electron gas. Highly Conducting One-Dimensional Solids.

[3] Brezin E., Zinn-Justin J. (1988). Field Theory Methods and Quantum Critical Phenomena.

[4] Stone M. (1994). Bosonization.

[5] Giamarchi T. (2003). Quantum Physics in One Dimension.

[6] Gogolin A.O., Nersesyan A.A., Tsvelik A.M. (2004). Bosonization and Strongly Correlated Systems.

[7] Jordan P., Wigner E. (1928). Über das Paulische Äquivalenzverbot. Z. Fur Phys..

[8] Lieb E., Schultz T., Mattis D. (1961). Two soluble models of an antiferromagnetic chain. Ann. Phys..

[9] Rodrigues W.A. (2017). Bosonization of Fermionic Fields and Fermionization of Bosonic Fields. Adv. Appl. Clifford Algebr..

[10] Seiberg N., Senthil T., Wang C., Witten E. (2016). A Duality Web in 2+1 Dimensions and Condensed Matter Physics. Ann. Phys..

[11] Izaurieta F., Rodríguez E., Salgado P. (2006). Expanding Lie (super)algebras through Abelian semigroups. J. Math. Phys..

[12] Concha P.K., Peñafiel D.M., Rodríguez E.K., Salgado P. (2013). Even-dimensional General Relativity from Born-Infeld gravity. Phys. Lett. B.

[13] Concha P.K., Durka R., Merino N., Rodríguez E.K. (2016). New family of Maxwell like algebras. Phys. Lett. B.

[14] Concha P.K., Durka R., Inostroza C., Merino N., Rodríguez E.K. (2016). Pure Lovelock gravity and Chern-Simons theory. Phys. Rev. D.

[15] González N., Rubio G., Salgado P., Salgado S. (2016). Generalized Galilean algebras and Newtonian gravity. Phys. Lett. B.

[16] Durka R. (2017). Resonant algebras and gravity. J. Phys. A.

[17] Concha P.K., Merino N., Rodríguez E.K. (2017). Lovelock gravities from Born–Infeld gravity theory. Phys. Lett. B.

[18] Ipinza M.C., Lingua F., Peñafiel D.M., Ravera L. (2016). An Analytic Method for *S*-Expansion involving Resonance and Reduction. Fortsch. Phys..

[19] Peñafiel D.M., Ravera L. (2017). Infinite S-Expansion with Ideal Subtraction and Some Applications. J. Math. Phys..

[20] Caroca R., Concha P., Rodríguez E., Salgado-Rebolledo P. (2018). Generalizing the ${\mathrm{bms}}_{3}$ and 2D-conformal algebras by expanding the Virasoro algebra. Eur. Phys. J. C.

[21] Caroca R., Concha P., Fierro O., Rodríguez E., Salgado-Rebolledo P. (2018). Generalized Chern–Simons higher-spin gravity theories in three dimensions. Nucl. Phys. B.

[22] Concha P., Merino N., Miskovic O., Rodríguez E., Salgado-Rebolledo P., Valdivia O. (2018). Asymptotic symmetries of three-dimensional Chern-Simons gravity for the Maxwell algebra. JHEP.

[23] Concha P., Merino N., Rodríguez E., Salgado-Rebolledo P., Valdivia O. (2019). Semi-simple enlargement of the ${\mathrm{bms}}_{3}$ algebra from a so(2,2)⊕so(2,1) Chern-Simons theory. JHEP.

[24] Bergshoeff E., Izquierdo J.M., Ortín T., Romano L. (2019). Lie Algebra Expansions and Actions for Non-Relativistic Gravity. JHEP.

[25] Concha P., Rodríguez E. (2019). Non-Relativistic Gravity Theory based on an Enlargement of the Extended Bargmann Algebra. JHEP.

[26] Durka R., Kowalski-Glikman J. (2019). Resonant algebras in Chern-Simons model of topological insulators. Phys. Lett. B.

[27] Concha P., Ravera L., Rodríguez E., Rubio G. (2020). Three-dimensional Maxwellian Extended Newtonian gravity and flat limit. JHEP.

[28] Concha P., Peñafiel D., Ravera L., Rodríguez E. (2021). Three-dimensional Maxwellian Carroll gravity theory and the cosmological constant. Phys. Lett. B.

[29] Concha P., Pino D., Ravera L., Rodríguez E. (2024). Extended kinematical 3D gravity theories. JHEP.

[30] Sohnius M.F. (1985). Introducing Supersymmetry. Phys. Rept..

[31] Castellani L., D’Auria R., Frè P. (1991). Supergravity and superstrings: A Geometric Perspective. Vol. 2: Supergravity.

[32] Alvarez P.D., Valenzuela M., Zanelli J. (2012). Supersymmetry of a different kind. JHEP.

[33] Alvarez P.D., Pais P., Zanelli J. (2014). Unconventional supersymmetry and its breaking. Phys. Lett. B.

[34] Guevara A., Pais P., Zanelli J. (2016). Dynamical Contents of Unconventional Supersymmetry. JHEP.

[35] Alvarez P.D., Delage L., Valenzuela M., Zanelli J. (2021). Unconventional SUSY and Conventional Physics: A Pedagogical Review. Symmetry.

[36] Nieh H.T., Yan M.L. (1982). Quantized Dirac Field in Curved Riemann-cartan Background. 1. Symmetry Properties, Green’s Function. Ann. Phys..

